# Tunable Microcavity-Stabilized Quantum Cascade Laser for Mid-IR High-Resolution Spectroscopy and Sensing

**DOI:** 10.3390/s16020238

**Published:** 2016-02-17

**Authors:** Simone Borri, Mario Siciliani de Cumis, Giacomo Insero, Saverio Bartalini, Pablo Cancio Pastor, Davide Mazzotti, Iacopo Galli, Giovanni Giusfredi, Gabriele Santambrogio, Anatoliy Savchenkov, Danny Eliyahu, Vladimir Ilchenko, Naota Akikusa, Andrey Matsko, Lute Maleki, Paolo De Natale

**Affiliations:** 1 CNR-INO – Istituto Nazionale di Ottica, Largo E. Fermi 6, 50125 Firenze, FI, Italy; m.siciliani@inrim.it (M.S.C.); insero@lens.unifi.it (G.I.); saverio.bartalini@ino.it (S.B.); pablo.canciopastor@ino.it (P.C.P.); davide.mazzotti@ino.it (D.M.); iacopo.galli@ino.it (I.G.); giovanni.giusfredi@ino.it (G.G.); g.santambrogio@inrim.it (G.S.); paolo.denatale@ino.it (P.D.N.); 2 LENS – European Laboratory for Non-Linear Spectroscopy, Via Carrara 1, 50019 Sesto Fiorentino, FI, Italy; 3 INFN – Istituto Nazionale di Fisica Nucleare, Sezione di Firenze, via G. Sansone 1, 50019 Sesto Fiorentino, FI, Italy; 4 INRIM – Istituto Nazionale di Ricerca Metrologica, Strada delle Cacce 91, 10135 Torino, Italy; 5 OEwaves Inc., 465 North Halstead Street, Suite 140, Pasadena, CA 91107, USA; Anatoliy.Savchenkov@oewaves.com (A.S.); Danny.Eliyahu@oewaves.com (D.E.); vladimir.Ilchenko@oewaves.com (V.I.); andrey.matsko@oewaves.com (A.M.); Lute.Maleki@oewaves.com (L.M.); 6 Development Bureau Laser Device R & D Group, Hamamatsu Photonics KK, Shizuoka 434-8601, Japan; aki@crl.hpk.co.jp

**Keywords:** sub-Doppler spectroscopy, quantum cascade lasers, laser stabilization, whispering gallery mode resonators, crystalline resonators, infrared resonator

## Abstract

The need for highly performing and stable methods for mid-IR molecular sensing and metrology pushes towards the development of more and more compact and robust systems. Among the innovative solutions aimed at answering the need for stable mid-IR references are crystalline microresonators, which have recently shown excellent capabilities for frequency stabilization and linewidth narrowing of quantum cascade lasers with compact setups. In this work, we report on the first system for mid-IR high-resolution spectroscopy based on a quantum cascade laser locked to a CaF${}_{2}$ microresonator. Electronic locking narrows the laser linewidth by one order of magnitude and guarantees good stability over long timescales, allowing, at the same time, an easy way for finely tuning the laser frequency over the molecular absorption line. Improvements in terms of resolution and frequency stability of the source are demonstrated by direct sub-Doppler recording of a molecular line.

## 1. Introduction

Narrow-linewidth frequency-stabilized lasers having mid-IR emission wavelengths have been demonstrated as the most suitable sources for both high-resolution and high-sensitivity molecular spectroscopy. The strong molecular rovibrational transitions that can be accessed with mid-IR laser sources have allowed trace-gas sensing down to the parts-per-quadrillion level [1] and frequency metrology on simple molecules, showing the potential for testing fundamental principles of physics at <1 eV energy scales [2,3,4,5,6]. Room temperature quantum cascade lasers (QCLs) are particularly attractive for these applications because they emit in the mid IR, with an mW to W optical power level and a wide spectral coverage. Although QCLs are characterized by sub-kHz intrinsic linewidth [7,8], their actual frequency stability is strongly affected by instrumental noise contributions such as current noise from the laser driver and temperature fluctuations [9]. A conventional QCL shows frequency tuning with temperature and current of the order of a few GHz/K and hundreds of MHz/mA, respectively. Typical drifts of several hundreds of MHz per hour can be expected, due to temperature fluctuations, and low-noise commercial current drivers can provide linewidths (over a ms timescale) from the 1–10 MHz range up to several tens of MHz. Therefore, frequency stabilization is important for sensing on pressure-broadened transitions, especially when long averaging is required, and it is an even more stringent requirement for high-resolution Doppler-free spectroscopy.

Various techniques can be used for laser frequency stabilization. Several years ago, it was demonstrated that even with a low-bandwidth locking loop, able to correct only slow fluctuations, significant improvements in precision spectroscopy with QCLs could be achieved [10]. More recently, QCLs have been locked to stable mid-IR references obtained via coherent frequency down-conversion, achieving sub-kHz linewidths [11,12]. Such stabilization technique allowed to get the best performances with QCLs, but, due to its extreme complexity, it is indeed not suitable for cost-effective and compact apparatuses, nor for field applications. The same problem affects QCL stabilization onto optical ultra-stable (ULE) cavities [13]. As such high-performing cavities work with visible or near-IR radiation, this method involves up-conversion of mid-IR lasers. QCLs locked to narrow molecular references can achieve sub-kHz linewidths [14]. However, a proper molecular transition must be available, and locking to a fixed reference makes a frequency tuning over a wide range more complex. Indeed, direct locking of QCLs to mid-IR cavities looks as the most practical stabilization method.

Searching for smart solutions to develop highly-integrated and compact sensors for mid-IR spectroscopic applications, QCL stabilization based on chip-scale microcavities appears as a natural solution. However, a standard Fabry–Perot resonator requires either a large cavity size to achieve a high quality factor (*Q*) or very high-reflectivity mirrors, which are not easily available at longer, infrared wavelengths. Instead, we recently demonstrated [15] that the use of a high-*Q* (∼$10^{7}$) monolithic crystalline whispering gallery mode resonator (WGMR) enables smart solutions and excellent performance.

Whispering gallery modes occur at particular resonant wavelengths depending on the size of the resonator. At these wavelengths, light is confined in the resonator for a long time by total internal reflection [16]. Crystalline microresonators have undergone an impressive development in the last decade, opening up new possibilities for photonic applications at the mm to μm dimensional scale. Although WGMRs are interesting physical objects themselves, they have several practical applications, including optical filters, modulators, lasers, optoelectronic silicon devices suitable for telecommunications [17]. Small volumes and high *Q*-factors (up to 10${}^{10}$ in the near-IR) of WGMRs result in enhancement of non-linear optical processes. The low threshold values for these processes (sometimes a few μW) have allowed for nonlinear generation of frequency combs in the near- and mid-IR region [18,19]. Thanks to their narrow modes, WGMRs have been used for frequency stabilization and linewidth narrowing of near-IR lasers by means of electronic or optical locking [20,21]. In our recent work [15], a WGMR made of CaF${}_{2}$ was tested for the first time for laser stabilization in the mid IR. It was injected by a QCL emitting at 4.3 μm wavelength, and both electronic locking on the transmission mode and optical self-injection locking were tested with excellent final laser stability (∼10 kHz linewidth over 1-s timescale).

In this work, we report on Doppler-free mid-IR spectroscopy using a QCL-resonator system, in electronic locking conditions. We show that the resonator mode frequencies can be finely tuned by acting on the resonator temperature. In this way, we demonstrate fine tuning of the locked laser over more than 1 GHz with no additional current or temperature feed-forward sent to the laser. The relative intensity noise (RIN) of the laser is shown both in free-running and locking conditions. Thanks to the linewidth reduction by more than one order of magnitude achieved with electronic locking, we show that improved sub-Doppler spectra can be recorded with the locked laser with respect to the free-running case.

## 2. Experimental Section

**Figure 1:**
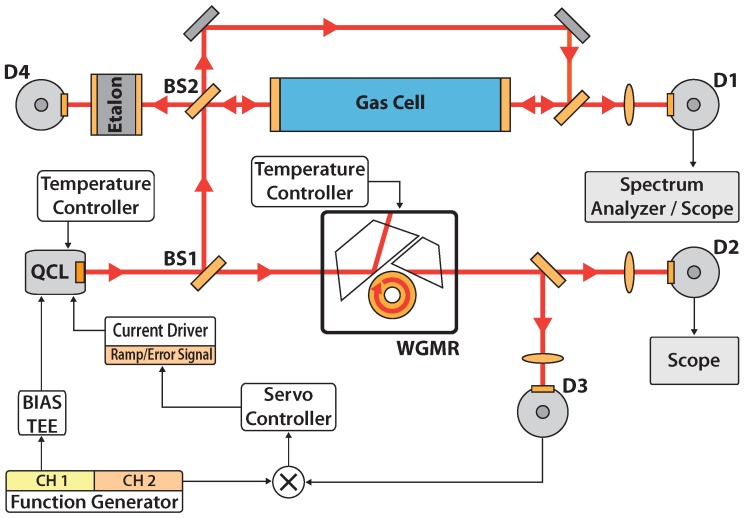
Schematic of the experimental setup used for electronic frequency stabilization. BS1, BS2: beam splitters. D1 and D2: InSb photodiodes; D3: HgCdTe photodiode; D4: PbSe photodiode.

A schematic of the experimental setup is shown in Figure 1. The source used in the experiment is a QCL operating at 4.3 μm wavelength by Hamamatsu Photonics. It is a single-mode distributed-feedback continuous-wave laser, operating at room-temperature (threshold current of about 700 mA at 15 ${}^{\circ }$C and maximum operating current ∼850 mA) and mounted in a home-made housing provided with a thermoelectric cooler and a collimating aspheric lens. A home-made current driver is used, provided with a modulation input (bandwidth from DC to about 1 MHz). A bias-tee mounted on the laser chip allows for fast modulations (up to tens of MHz).

The laser was operated at about 780 mA, with total emitted power slightly higher than 10 mW. As shown in Figure 1, the laser beam is split into two arms by a 50:50 beam splitter (BS1). The transmitted light is coupled to a OEwaves CaF${}_{2}$ toroidal WGMR (3.6 mm diameter). This resonator has a free-spectral range (FSR) of 18.9 GHz at the operating wavelength. It is mounted inside a custom-made housing to reduce both mechanical and thermal fluctuations and to protect it from dust and humidity. To allow free-beam evanescent-wave coupling, a sapphire prism is placed close to the resonator surface. The prism–WGMR gap depends on the temperature at which all the system is maintained. The transmission from the resonator is used for electronic locking by means of a thermoelectrically-cooled fast HgCdTe photodiode (Vigo PVI-4TE-5, ∼10 MHz bandwidth). This signal from the photodiode is processed by the locking electronics (frequency mixer and servo controller) and added to the laser current via the driver modulation input as a correction signal, as described in [15]. A liquid-N${}_{2}$-cooled InSb photodiode (Hamamatsu P5968-100, D2 in the figure) is also used for monitoring the transmitted signal in both unlocked and locked conditions.

The beam reflected from the beam-splitter is sent to the spectroscopic cell (10 cm length), filled with pure CO${}_{2}$ at up to ∼0.1 mbar pressure, in a standard pump-probe arrangement for Doppler-free detection. An uncoated CaF${}_{2}$ window is used as a beam splitter (BS2), providing a pump with about 5 mW radiation power (transmitted through the window) and a probe with about 0.3 mW (reflected beam). The pump and probe beams, which counterpropagates within the cell, are slightly crossed in order to avoid a strong feedback onto the laser. The probe is detected by a liquid-N${}_{2}$-cooled InSb photodiode (Hamamatsu P5968-060, D1 in the figure). In order to measure the tuning range, a Ge etalon (thickness 3.75 cm, 1 GHz FSR ) is used as reference, as shown in the figure.

**Figure 2:**
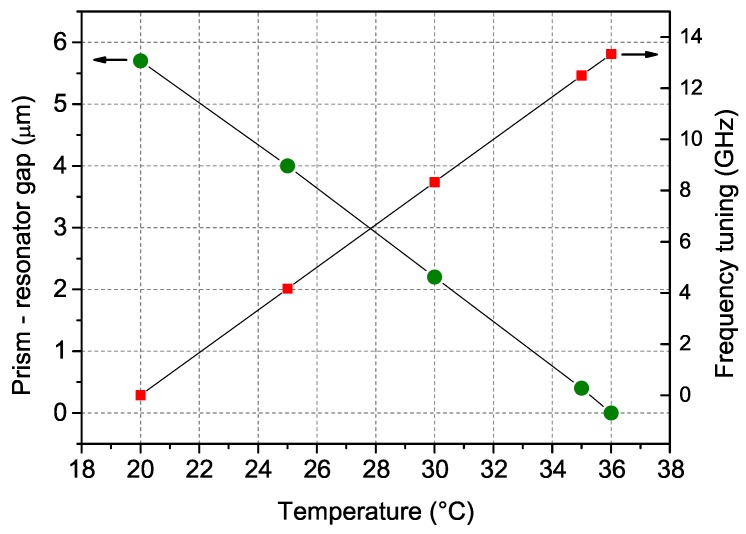
Temperature dependence for the coupling prism-WGMR gap (left scale), measured interferometrically; and for the WGMR mode frequency tuning (right scale).

As mentioned above, tuning the temperature of the resonator has a twofold effect. On one hand, it modifies the coupling prism–WGMR gap and, as a consequence, the amount of light coupled into the resonator: This translates into a change of the WGMR finesse (*Q*-factor) and of the mode width. On the other hand, it also changes the resonator dimensions and refractive index, allowing for fine frequency tuning of the transmission mode. The measured tuning coefficient with temperature is about 0.85 GHz/K. This behavior is reported in Figure 2.

**Figure 3:**
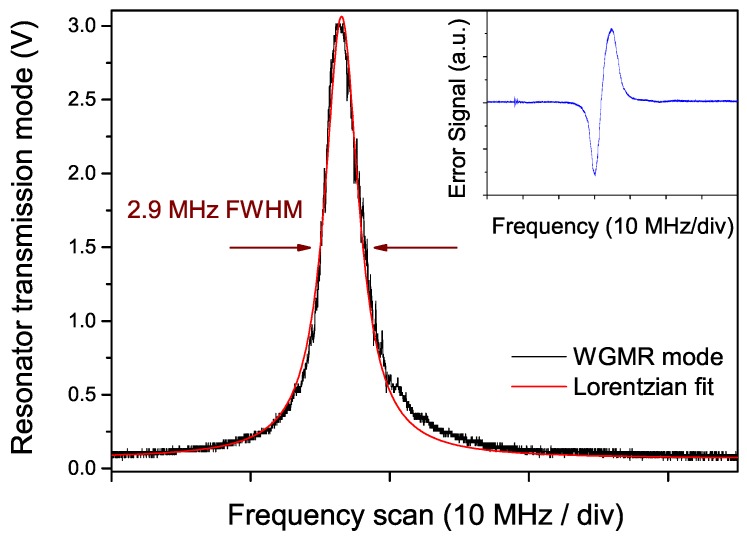
Resonator transmission mode corresponding to a temperature of ∼34.5 ${}^{\circ }$C. The trace was acquired with a scan time duration of 50 ms. The fitting Lorentzian curve is shown in red. Inset: error signal at the mixer output (modulation frequency 3.5 MHz, modulation depth ∼5 MHz).

Figure 3 shows the profile of the resonator mode (transmission) at the operating temperature of 34.5 ${}^{\circ }$C. In these conditions, the WGMR mode is in resonance with a strong CO${}_{2}$ line, the (00${}^{0}$1–00${}^{0}$0) P(42) transition lying at 2311.105 cm${}^{-1}$, having a linestrength of 4.75 × 10${}^{-19}$ cm (in HITRAN units).

In the figure, a Lorentzian profile is fitted to the recorded mode, showing a good agreement. The slight distortion visible at one side (right in the figure) reflects thermal effects occurring during a frequency scan of a high-*Q* resonator mode. The mode width is about 2.9 MHz (FWHM), corresponding to a *Q*-factor of 2.3 ×$10^{7}$ (finesse F≃ 6300).

## 3. Noise Analysis

An analysis of the laser frequency-noise power spectral density (FNPSD) has been carried out, both in free-running and locking conditions. A waveform generator was used to add a slow triangular ramp (100 ms period) to the modulation input of the current driver to scan the frequency through the resonator modes and through the absorption line. In order to generate the error signal, a frequency-modulation-like scheme was implemented: a fast sinusoidal modulation was generated by a dual-channel function generator and sent to the laser via the external bias-tee. The transmission from the resonator detected by the fast photodiode was demodulated by means of a mixer and processed by the servo electronics, consisting of a proportional and an integral (PI) stage, and finally sent as feedback signal to the laser current via the driver modulation input port. The error signal at the mixer output is shown in the inset of Figure 3. The selected values for the modulation frequency and depth were 3.5 MHz and ≃5 MHz, respectively.

The FNPSD was measured by using the side of the molecular absorption line as frequency-to-amplitude converter, under relatively high-pressure conditions (∼2 mbar). For the noise analysis, the detector D1 (bandwidth ∼300 kHz) and a real-time spectrum analyzer (Tektronix RSA 3303A, DC-3 GHz bandwidth) were used.

**Figure 4:**
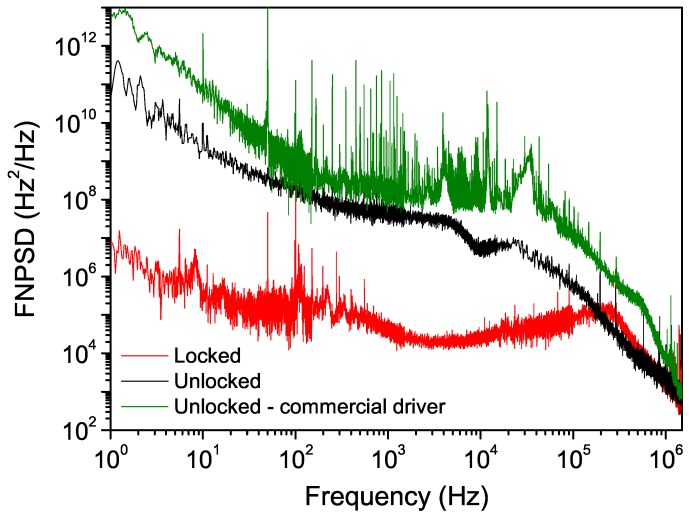
Frequency noise spectral power density of the laser in both free-running and locking conditions (black and red traces). The FNPSD with the laser operated with a commercial driver is also shown for comparison (green trace).

The black trace in Figure 4 shows the unlocked laser FNPSD. An integral of the noise curve following the method in [22] gives a laser linewidth of about 2.2 MHz FWHM over a 1-s timescale. Locking the laser to the resonator led to a noise reduction of more than three orders of magnitude up to about 10 kHz, with an overall correction bandwidth of about 200 kHz. We achieved an overall linewidth reduction down to about 200 kHz over 1 s, about a factor of ten with respect to the unlocked case. With respect to our previous work [15], we had to reduce both the bandwidth of the integral stage and the gain of the proportional one in order to allow for a wide tuning without losing the locking condition.

For comparison, the laser FNPSD also obtained using a commercial current driver is shown (green trace). This driver has the advantage of high compliance and supplied current (up to 4 A), but it is affected by a high current noise. With this driver, an integral of the FNPSD gives a linewidth larger than 16 MHz FWHM over the same 1-s timescale. Moreover, the transmission from the resonator (and, consequently, the error signal) was too noisy for a stable locking.

**Figure 5:**
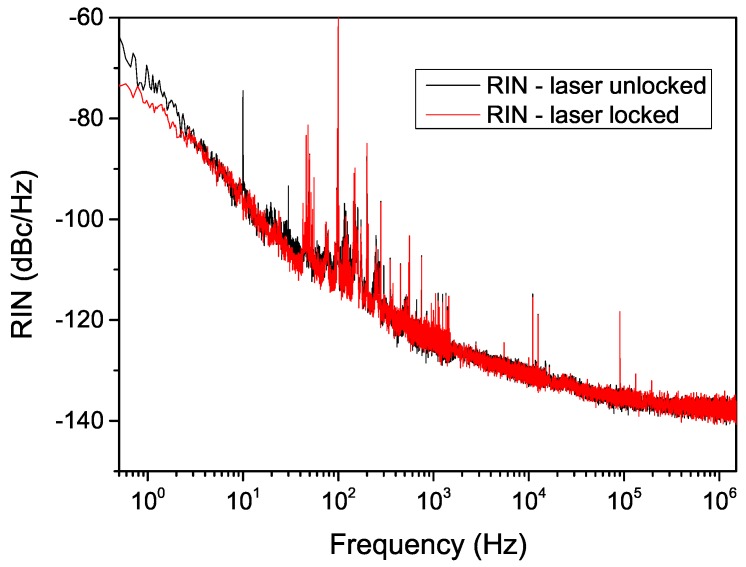
RIN spectral distribution in locked and unlocked conditions.

By moving the laser far away from the absorption line, we also measured the relative intensity noise (RIN) spectral distribution in locked and unlocked conditions (with the home-made driver). As shown in Figure 5, no significant variation can be observed between the two cases, apart from a slight noise reduction in the very low frequency part of the spectrum (below 10 Hz).

## 4. Spectroscopic Results

**Figure 6:**
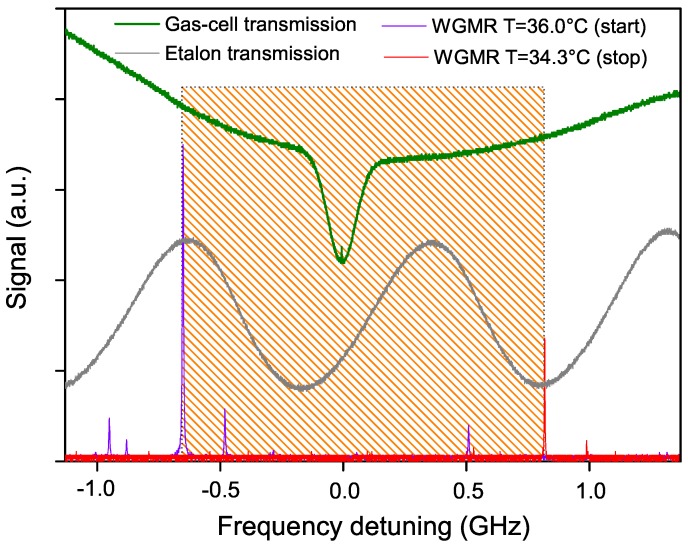
Representative frequency scan of the laser showing the tuning range in locking condition across the molecular transition. The horizontal scale represents the frequency detuning from the transition center (69285185(3) MHz as given by HITRAN). The green trace is the signal transmitted through the spectroscopic cell, and shows the molecular line (Doppler profile over pressure-broadened absorption from air); the purple and red traces are the main resonator modes to which the laser is locked, at the starting and ending points of the frequency scan; the gray trace shows the signal transmitted through the Ge etalon (1 GHz FSR). The hatched region shows the continuous tuning range achieved in locking condition only by acting on the resonator temperature.

When the stabilization loop is closed, a fine tuning of the WGMR resonance up to about 1.5 GHz can be achieved without losing the locking condition, by acting only on its temperature. It is worth noting that, in this proof-of-principle demonstration, no additional current or temperature feed-forward was sent to the laser. In Figure 6, the green trace shows the absorption profile after the cell (CO${}_{2}$ pressure about 0.1 mbar). The effect of the absorption from the ambient CO${}_{2}$ is evident (broad parabolic-like shape in the green trace). The Doppler-broadened absorption due to the low-pressure CO${}_{2}$ in the cell emerges from the background and the Lamb dip can be distinguished at its center. The continuous tuning range achieved in the locking condition is represented in the figure by the hatched region. The WGMR transmission modes recorded at the initial and final temperatures are shown in violet and red, respectively. The gray sinusoidal-like trace is the etalon transmission (1 GHz FSR).

A strong difference is evident from Figure 6 in the resonator mode amplitude between the initial and final points of the frequency scan. This is due to the difference in temperature that leads to different light coupling levels, as explained in Section 2. This imposes a limit on the tightness of the lock to the resonator: the correction signal changes following the change in the resonator mode amplitude (and width), thus intermediate values for the PI stages must be chosen in order to maintain stable locking conditions throughout the frequency scan. It is worth noting that this limit can be overcome by adopting an active compensation of the feedback signal, which could lead not only to a narrower linewidth but also to a wider tuning range. Nonetheless, a frequency tuning was achieved that is broad enough to span Doppler-broadened transitions. We also have to note that resonators with *Q* unaffected by the mode frequency (and thus by the temperature) can be developed, which may be very advantageous for spectroscopy when wide tuning ranges are required.

**Figure 7:**
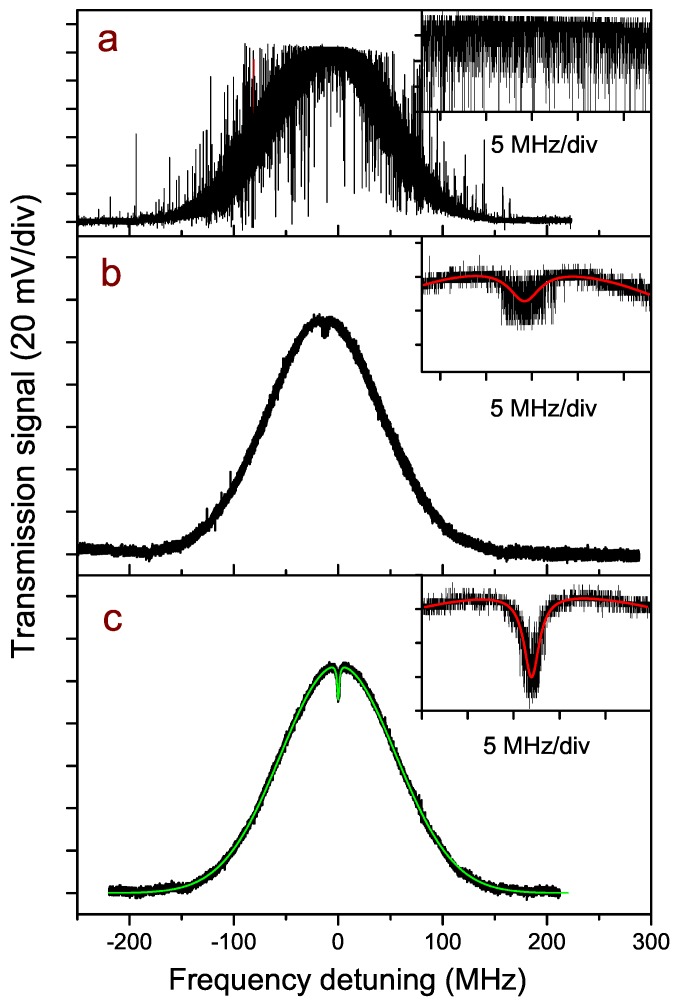
Optical transmission signal through the spectroscopic cell with the laser scanning of the CO${}_{2}$ transition. The three cases corresponding to the different traces are: free-running laser operated with the commercial driver (**a**); laser operated with the home-made driver in free-running (**b**) and locking (**c**) conditions. All three graphs have the same horizontal scale, representing the detuning from the center frequency of the transition. In each inset, a zoom of the top line profile is shown, over the same horizontal and vertical scales, with the red curves showing the Lorentzian fit of the dip (**b**,**c**). The thin green trace superimposed on the data in (**c**) is a simulation of the sub-Doppler feature based on the HITRAN data, as described in the text.

In order to demonstrate the possibility of high-resolution spectroscopic measurements, and to point out the improvements given by the linewidth narrowing, we performed sub-Doppler measurements with a pump-probe scheme. Figure 7 summarizes the results in three different conditions, with a CO${}_{2}$ pressure of 0.1 mbar in the cell. For all of the three traces, the total scan time duration was 40 s. The background due to air absorption and to residual amplitude modulation of the laser (about 2% intensity variation over 1 GHz frequency span) was fitted and removed from the spectra.

When the commercial driver was used, no Lamb dip can be observed due to the large laser linewidth. Figure 7a shows a scan over the molecular line (transmission from the cell) recorded in this condition, and, in the inset, a zoom around the line peak is shown. Figure 7b,c show, over the same horizontal scale, the CO${}_{2}$ line profile with the laser supplied by the home-made driver and operated in free-running and locking conditions, respectively. This time, the Lamb dip is clearly visible in both cases but, as better shown in the inset, in a locked regime, the dip is narrower and the contrast much higher (the vertical scale extension in the three insets is the same). A Lorentzian shape over the Doppler-broadened Gaussian profile is fitted to the experimental data (inset of the figures, red curves) and the results for the dip width are 4.1 MHz and 2.0 MHz (FWHM) respectively, in agreement with the difference in the laser linewidth measured in locking and free-running conditions. The 2.0 MHz FWHM is consistent with the broadening contributions due to the experimental conditions, given by a pressure broadening of about 400 kHz, the measured laser linewidth and a residual Doppler broadening due to the slight crossing (≤1${}^{\circ }$ angle) of the pump and probe beams. Improvements can be observed also in terms of the dip contrast, sensibly better in locking condition with respect to the free-running case. The fit shows that an improvement by more than a factor of three is obtained even on the dip center frequency determination, in locked with respect to unlocked laser, with absolute uncertainties of 9 kHz and 33 kHz, respectively. This method is expected to show all its potentiality when the integration time grows from the present few seconds to hours or days. In this case, in fact, we can take advantage of a high long-term relative frequency stability, which for a properly packaged WGMR has been measured to be better than 10${}^{-10}$ per day [21]. Finally, in order to compare the recorded saturated spectrum with the HITRAN data, in Figure 7c, a simulation of the saturated line is shown (thin green curve) by considering the measured linewidth of 2 MHz and the HITRAN data for the selected transition. In particular, a saturation parameter ∼0.6 is assumed, which is a reasonable estimation from our experimental parameters. We note a good agreement between this simulated HITRAN spectrum and the experimental one.

## 5. Conclusions

In conclusion, a compact apparatus for sub-Doppler spectroscopy based on a mid-IR QCL locked to a high-*Q* CaF${}_{2}$ microresonator has been reported. This system, reported here as a proof-of-principle, is able to stabilize the laser frequency and to narrow its linewidth offering, at the same time, a wide and easy tuning capability. Laser fine tuning in locking condition up to 1.5 GHz was obtained by acting only on the resonator temperature, without any other active correction on the laser current and temperature. By slightly refining the locking mechanism, in particular by correcting the change in the error signal shape which occurs during the temperature scan, better performance in both linewidth reduction and tuning range is expected. Nonetheless, even with present limitations, the good stability over long timescales of the system (obtained without any need for locking the resonator to a frequency reference), its wide tunability, its compactness and the overall simplicity put it among the most promising systems for high-resolution molecular spectroscopy with narrow-linewidth mid-IR sources.
